# Comparison of lipooligosaccharides from human challenge strains of *Neisseria gonorrhoeae*

**DOI:** 10.3389/fmicb.2023.1215946

**Published:** 2023-09-15

**Authors:** Constance M. John, Nancy J. Phillips, Amaris J. Cardenas, Alison K. Criss, Gary A. Jarvis

**Affiliations:** ^1^Veterans Affairs Medical Center, San Francisco, CA, United States; ^2^Department of Laboratory Medicine, University of California, San Francisco, San Francisco, CA, United States; ^3^Department of Pharmaceutical Chemistry, University of California, San Francisco, San Francisco, CA, United States; ^4^Department of Microbiology, Immunology, and Cancer Biology, University of Virginia, Charlottesville, VA, United States

**Keywords:** glycolipids, inflammation, lipid A, mass spectrometry, *Neisseria gonorrhoeae*, phosphoethanolamine, phospholipids

## Abstract

The alarming rise of antibiotic resistance and the emergence of new vaccine technologies have increased the focus on vaccination to control gonorrhea. *Neisseria gonorrhoeae* strains FA1090 and MS11 have been used in challenge studies in human males. We used negative-ion MALDI-TOF MS to profile intact lipooligosaccharide (LOS) from strains MS11mkA, MS11mkC, FA1090 A23a, and FA1090 1-81-S2. The MS11mkC and 1-81-S2 variants were isolated from male volunteers infected with MS11mkA and A23a, respectively. LOS profiles were obtained after purification using the classical phenol water extraction method and by microwave-enhanced enzymatic digestion, which is more amenable for small-scale work. Despite detecting some differences in the LOS profiles, the same major species were observed, indicating that microwave-enhanced enzymatic digestion is appropriate for MS studies. The compositions determined for MS11mkA and mkC LOS were consistent with previous reports. FA1090 is strongly recognized by mAb 2C7, an antibody-binding LOS with both α- and β-chains if the latter is a lactosyl group. The spectra of the A23a and 1-81-S2 FA1090 LOS were similar to each other and consistent with the expression of α-chain lacto-*N*-neotetraose and β-chain lactosyl moieties that can both be acceptor sites for sialic acid substitution. 1-81-S2 LOS was analyzed after culture with and without media supplemented with cytidine-5'-monophosphate *N*-acetylneuraminic acid (CMP-Neu5Ac), which *N. gonorrhoeae* needs to sialylate its LOS. LOS sialylation reduces the infectivity of gonococci in men, although it induces serum resistance in serum-sensitive strains and reduces killing by neutrophils and antimicrobial peptides. The infectivity of FA1090 in men is much lower than that of MS11mkC, but the reason for this difference is unclear. Interestingly, some peaks in the spectra of 1-81-S2 LOS after bacterial culture with CMP-Neu5Ac were consistent with disialylation of the LOS, which could be relevant to the reduced infectivity of FA1090 in men and could have implications regarding the phase variation of the LOS and the natural history of infection.

## Introduction

*Neisseria gonorrhoeae* is an exclusively human pathogen that causes the sexually transmitted disease gonorrhea. In 2020, the WHO estimated that approximately 82 million cases of gonorrhea occur worldwide annually. The CDC estimated that there were 1.6 million cases of gonorrhea in the United States in 2018, more than half of those between 15 and 24 years of age (Kreisel et al., 2021). The bacteria are increasingly multi-drug resistant, with high rates of resistance to antibiotics including azithromycin and extended-spectrum cephalosporins which are considered to be last resort treatments (Unemo et al., 2021). *N. gonorrhoeae* readily undergo antigenic variation, and the limited immune responses and lack of protection against reinfection have hampered the successful development of a vaccine. However, the alarming rise of antibiotic resistance and the emergence of new vaccine technologies have increased the focus on vaccination to control the disease in the future.

*N. gonorrhoeae* infects mucous membranes of the mouth, throat, eyes, and rectum, and the reproductive tract—the cervix, uterus, and fallopian tubes in women and the urethra in both women and men. However, the pathobiology of gonococcal infections differs between men and women (Edwards and Butler, 2011). The frequency of asymptomatic and subclinical urogenital infections is higher in women. Rectal and pharyngeal infections are usually asymptomatic in both men and women (Dombrowski, 2021). Experimental infection of women with *N. gonorrhoeae* is unsafe due to the potential for ascension of bacteria into the upper urogenital tract, whereas a controlled human male urethral infection model that replicates the natural course of the disease has been safely used over the last 3 decades to study pathogenicity and immunity in several hundred subjects who were inoculated with strains FA1090 and MS11 (Cohen et al., 1994; Hobbs and Duncan, 2019; Waltmann et al., 2022). These strains have also been used to examine murine antisera reactivity to the 4CMenB meningococcal recombinant protein-detoxified outer membrane vesicle vaccine (Leduc et al., 2020) and preclinical testing of an LOS peptide mimetic vaccine (Gulati et al., 2019). Mutant and wild-type FA1090 bacteria were used in experimental infections with mixtures of wild-type *N. gonorrhoeae* and isogenic mutants that lacked the enzyme EptA, which transfers phosphoethanolamine (PEA) onto lipid A, which showed that EptA expression conferred a significant survival advantage *in vivo* (Hobbs et al., 2013).

Multiple logistic regression analyses of human studies conducted using FA1090 and MS11 revealed that although the inocula of both strains were primarily opacity protein negative (Opa-) and piliated (P+) and significant efforts were made to control phase-variable determinants, the infection of 50% of subjects (ID_50_) required a 55-fold lower inoculum of MS11mkC than FA1090 A23a (Hobbs et al., 2011). MS11mkC, unlike FA1090, expresses the lactoferrin-binding proteins A and B and produces higher amounts of the MtrC-MtrD-MtrE multidrug efflux pump, which confers resistance to antimicrobial drugs by transporting them out of the gonococcus and expresses a type IV secretion system (Biswas and Sparling, 1995; Hamilton et al., 2005; Warner et al., 2008; Ohneck et al., 2011). Nonetheless, FA1090 is highly serum-resistant, whereas MS11mkC is moderately serum-sensitive (Carbonetti et al., 1990; Chen and Seifert, 2013). Despite these data showing differences between the two strains, the reason for the dramatic variation between them in male infectivity remains unclear (Hobbs et al., 2011).

There is compelling evidence that the outer membrane LOS is a virulence factor and that the modulation of the structure of the LOS by gonococci alters their interactions with the host in a manner that is likely to facilitate bacterial infection and survival (Post et al., 2002; Wu and Jerse, 2006; Johnson and Criss, 2013). There have been several reports of analysis of the LOS structure of variants of the MS11 strain (Kerwood et al., 1992; John et al., 1999; Minor et al., 2000), but only immunoblot data and monosaccharide compositional analyses have been reported for FA1090 LOS (Erwin et al., 1996; Hobbs et al., 2013). Both MS11mkC (John et al., 1999) and FA1090 (Hobbs et al., 2013) express the lacto-*N*-neotetraose (LNnT Galβ1-4GlcNAcβ1-3Galβ1-4Glcβ1) LOS epitope that is recognized by the 3F11 mAb (Yamasaki et al., 1994; Erwin et al., 1996). Among 8 strains of *N. gonorrhoeae*, FA1090 and 15323 were most strongly recognized by the 2C7 mAb (Erwin et al., 1996), whereas it does not bind LOS such as MS11mkC lacking a B-chain lactosyl group (Banerjee et al., 1998). The 2C7 epitope has been defined as gonococcal LOS with the γ-chain GlcNAc, an α-chain with an initial or only a lactosyl group, and a β-chain with only a lactosyl group (Yamasaki et al., 1999). The oligosaccharide of the gonococcal LOS is subject to phase variation depending on in- or out-of-frame mispairing of polymeric guanine tracts occurring within some of the glycosyltransferase *(GTase)* genes during replication (Gotschlich, 1994; Jordan et al., 2005; McLaughlin et al., 2012; Chakraborti et al., 2016).

Our ultimate goal is to compare the structures of the LOS based on our hypothesis that differences in the LOS structures could be the major reason for the lower infectivity of the FA1090 strain compared to MS11mkC. Among the LOS substituents that have been shown to affect virulence and infectivity are the phosphorylation and phosphoethanolaminylation of lipid A. The relative inflammatory potential (John et al., 2009b; Liu et al., 2010; Kahler et al., 2018; Samantha and Vrielink, 2020), sensitivity to complement and endogenous antimicrobial peptides (Kerwood et al., 1992; Lewis et al., 2009, 2013), and bacterial fitness (Hobbs et al., 2013) have all been correlated with differential expression of lipid A phosphoforms.

*N. gonorrhoeae* does not produce CMP-Neu5Ac, but during infection, the bacteria can utilize CMP-Neu5Ac from its human host to sialylate its LOS. Previous analyses have indicated that the gonococcal sialyltransferase (Lst) enzyme can add sialic acid to multiple acceptors on the LOS. Sialic acid has been found in an α(2,3)-linkage to the terminal tetrasaccharide, LNnT, and an α(2,6)-linkage to the alternate terminal P^*k*^-trisaccharide, Galα1-4Galβ1-4Glcβ1, which are linked to heptose I (HepI) and, furthermore, via an α(2,6)-linkage to a terminal disaccharide lactose, Galβ1-4Glcβ1, on HepII (Ram et al., 1998; Gulati et al., 2005). One mechanism whereby gonococci inhibit complement-mediated phagocytosis is through sialylation of LNnT LOS that then can bind to the complement inhibitor human Factor H, which decreases the binding of C3 fragments in a process that is dependent on expression of the porin subtype B1A (Kim et al., 1992; Jarvis, 1994; Ram et al., 1998; Ngampasutadol et al., 2008; Welsch and Ram, 2008).

Although a body of data has shown that sialylation of the LOS can reduce complement-mediated bacterial lysis and thereby enhance bacterial survival, the sialylation of LOS on MS11mkC bacteria by *in vitro* culture on media supplemented with CMP-Neu5Ac significantly reduced infectivity for human male volunteers (Schneider et al., 1996). The effect of sialylation on infectivity could be due to a reduction in endocytosis of the gonococci by the asialoglycoprotein receptor of human urethral epithelial cells (Harvey et al., 2001).

Purification of LOS from *N. meningitidis* and *N. gonorrhoeae* has been based on the classical method for phenol-water extraction (PE) following enzymatic digestions that was first described decades ago (Westphal and Jann, 1965; Johnson et al., 1975). Our ultimate goal is to directly analyze the phenotype and sialylation of LOS on gonococci isolated from infected humans and their cells, which would require methods enabling highly sensitive purification and detection. A method was described recently enabling LC-MS/MS of multiple LOS phenotypes from *N. meningitidis* with less than 1-μg LOS (Pupo et al., 2021). Herein, we evaluated a method for purification of the *N. gonorrhoea*e LOS using microwave-enhanced enzymatic (ME) digestions that was described for micro-scale purification of LOS from *Campylobacter jejuni* LOS and that shortened the time for LOS purification from 3–4 days to a few hours (Dzieciatkowska et al., 2008). Successful adaptation of the microwave-enhanced method for purification together with the sensitive methods for LC-MS/MS of LOS or our own methods for MALDI-TOF MS to the LOS from *N. gonorrhoeae* should enable us to accomplish our goal.

## Materials and methods

### Bacterial strains

**Figure 1:**
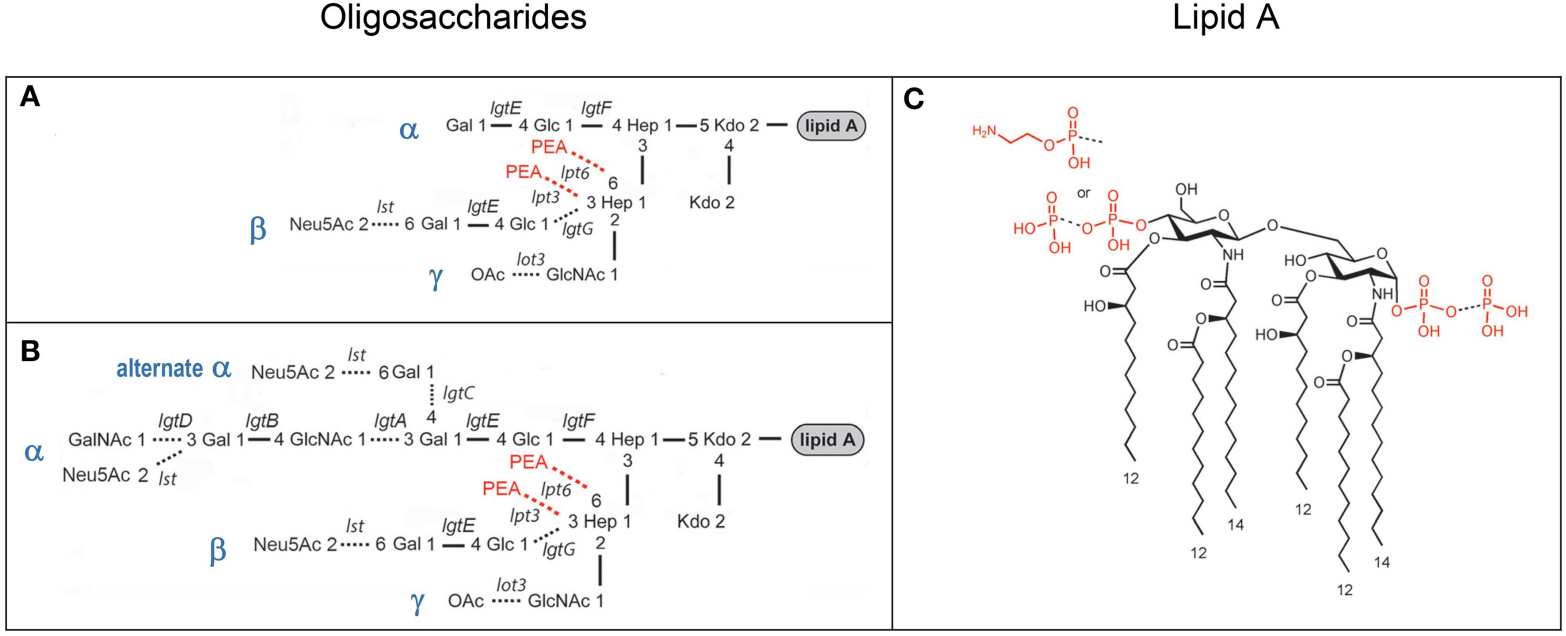
Composite of prototypical structures of the LOS of *N. gonorrhoeae* that illustrates the variable oligosaccharide α-chains on HepI and the β- and γ-chains on HepII **(A, B)** with the glycosyltransferases catalyzing the formation of the glycosidic bonds, and the conserved lipid A **(C)** with its variable phosphoryl substitution.

Experimental human studies of gonococcal infections over the last 25 years have used either MS11 or FA1090 strains of *N. gonorrhoeae*. MS11 is a strain of porin serotype PIB-9 that was isolated in 1970 from a patient with anterior urethritis (Edwards et al., 1984; Swanson et al., 1985). The variant A strain of MS11 is a descendent of MS11 that was used in a human study from which the variant C strain was isolated (Schneider et al., 1991). The MS11mkC variant strain expresses a LNnT α-chain moiety that can be substituted with one or more *N*-acetyllactosamine groups, whereas the α-chain of the MS11mkA variant is a lactose moiety (see Figure 1) (Kerwood et al., 1992; John et al., 1999). FA1090 A23a, a porin serotype PIB-3 strain, was isolated in the 1970s from the endocervix of a patient who likely had a disseminated infection (Nachamkin et al., 1981) and has been used in a number of human challenge studies (Cohen et al., 1994; Thomas et al., 2006; Hobbs et al., 2013). The 1-81-S2 variant strain was isolated in one of the human challenge studies of FA1090 A23a (Seifert et al., 1994). We also analyzed LOS prepared from an *eptA* mutant that we created from the 1-81-S2 isolate that we transformed with a plasmid containing insertionally inactivated *eptA* as previously described (Handing and Criss, 2015).

### LOS purification using the classical PE method

*N. gonorrhoeae* were grown on GC agar plates (100 mM diameter) containing Isovitalex (Becton-Dickinson). LOS was isolated based on a modification of the phenol-chloroform enzyme-digestion (PE) method (Apicella et al., 1994). Briefly, bacteria were gently scraped from 4–5 GC agar plates into a 50-ml centrifuge tube using phosphate-buffered saline (PBS) as needed. The supernatant was aspirated after pelleting the bacteria by centrifugation. Then, the pellet was suspended in 25 ml of 50 mM sodium phosphate, pH 7.0, containing 5 mM EDTA, by vortexing. Hen egg lysozyme (#L6876, Sigma-Aldrich, St. Louis, MO) was added to achieve a final concentration of 4 mg/ml, and the suspension was stirred overnight at 4°C. The next morning, the suspension was heated to 37°C for 20 min and then stirred well. After transferring to a 250-ml centrifuge bottle, the volume was increased to 100 ml with 50 mM sodium phosphate at pH 7.0 with 20 mM MgCl_2_. Next, DNaseI (Type IV from bovine pancreas, #D5025 Sigma-Aldrich) and RNase (Type III-A from bovine pancreas, #R5125, Sigma-Aldrich) were added to make a final concentration of 1 mg/ml each, and the suspension was incubated at 37°C for 60 min and then at 60°C for another 60 min. In the final digestion step, proteinase K (from *Tritirachium album*, #P2308, Sigma Aldrich) was added to a final concentration of 20 μg/ml, and the suspension was incubated at 50°C for 60 min. After heating the suspension in a water bath at 70°C for 10 min, 100 ml of 90% phenol preheated to 70°C was added. This mixture was rapidly cooled by placing it in an ice-water bath and then stirring for 15 min. The suspension was centrifuged at 18,000 × *g* for 15 min. A sharp interface formed between the bottom phenol and the top aqueous layer containing the LOS, which was removed using a 25-ml glass serological pipette. The aqueous phase was placed in a 1,000 MWCO membrane and dialyzed in deionized water at 4°C with 3–4 changes of water over a 1.5–2 day period until there was no detectable phenol odor. The dialyzed suspension of LOS was frozen and then lyophilized. Finally, deionized water was again added to the LOS (10–20 mg/ml), and the suspension was vortexed and/or sonicated to obtain a smooth suspension. The suspension was centrifuged at 100,000 × *g* for 4 h at 4°C. After discarding the supernatant, the insoluble pellet containing the LOS was resuspended in the original volume of deionized water, frozen, and then lyophilized.

### LOS purification with ME enzymatic digestion

Gonococcal LOS was purified using ME enzymatic digestion based on a method described for *Campylobacter jejuni* LOS (Dzieciatkowska et al., 2008) that entirely omits digestion with lysozyme and shortened the time required overall from 3–4 days to 1–2 h. *N. gonorrhoeae* were gently scraped from one-half to a whole GC agar plate (100 mM diameter) and suspended in 150–300 μL of 20 mM ammonium acetate buffer (pH 7.5) containing DNase (100 μg/ml) and RNase (200 μg/ml) in a 1.5- or 2.0-ml microcentrifuge tube that was securely closed and placed in a rack. The sample was heated in a 1,250-W microwave oven (Panasonic, Newark, NJ, United States) with a power setting of “2” for 3 min. Excessive energy was absorbed by setting a 100-ml glass beaker containing water near the rack. Then, an aliquot of proteinase K in solution in 20 mM ammonium acetate, pH 7.5, was added to produce a final concentration of 60 μg/ml and heated in the microwave as described above. After cooling to room temperature, the suspensions were frozen and lyophilized or dried on a Savant SpeedVac (Thermo Fisher Scientific, Waltham, MA, United States). The dried LOS samples were washed twice with methanol (300 μL) and twice with ethanol (300 μL) by vortexing, followed by centrifugation. Some samples were also dialyzed using 2000 MWCO Mini Dialysis Slide-A-Lyzer Units (Thermo Fisher Scientific).

### Preparation of intact LOS for MALDI-TOF MS

Intact LOS samples were prepared for MS analysis using modifications of a method described previously (Sturiale et al., 2005; John et al., 2009b). Briefly, purified LOS (4–10 mg/ml) was suspended in a methanol-water (1:3) solution with 5 mM EDTA, and an aliquot was desalted with a few cation-exchange beads (Dowex 50WX8-200) that had been converted to the ammonium form. A spot containing a layer of matrix was formed by deposition of 1 or 2 drops (~1.0 μL each) of a solution composed of 2,4,6-trihydroxyacetophenone (THAP; 200 mg/ml; Sigma-Aldrich, St. Louis, MO) in methanol with a nitrocellulose transblot membrane (15 mg/ml; Bio-Rad, Hercules, CA, United States) in acetone-isopropanol mixed in a 4:1 (v/v) ratio within inscribed circles on the stainless steel sample plate. The nitrocellulose membrane was solubilized in the acetone-isopropanol solution (1:1, v/v) with vigorous vortexing. The desalted sample solution was mixed with 100 mM dibasic ammonium citrate (9:1, v/v), and 1.0–2.0 μL was deposited on top of spots of the dried matrix.

### Hydrogen fluoride treatment of LOS

Phosphodiesters were partially removed by HF treatment. Native LOS (10 mg/ml) was reacted with 48% aqueous HF at 4°C for 16–20 h. Excess HF was removed using a Savant SpeedVac (Thermo Fisher Scientific) with an in-line trap containing sodium hydroxide pellets.

### MALDI-TOF MS

High-resolution MALDI-TOF MS analyses were performed on a Synapt G2 HDMS system (Waters Corporation, Milford, MA, United States) in “sensitivity mode” as previously described (Phillips et al., 2016). Spectra were obtained in negative-ion mode by operating a neodymium-doped yttrium aluminum garnet laser at 355 nm and 200 Hz. The masses of (M-H)^−^ monoisotopic ions for angiotensin II, renin substrate, and intact and B-chain bovine insulin (all from Sigma-Aldrich) were used for the calibration of the high-resolution analyses on the Synapt G2 HDMS.

We also performed low-resolution MALDI-TOF MS in the negative-ion linear mode on the Axima (Shimadzu Corp., Kyoto, Japan). The Axima spectra were calibrated in the “combical” mode using average (M-H)^−^ masses for 2–3 peaks for the lipid A prompt fragment ions along with the average masses of angiotensin II, renin substrate, and intact and B-chain bovine insulin, which had been analyzed in a separate spectrum.

## Results

### High-resolution MALDI-TOF MS profiling of MS11mkA LOS

Studies of the structure of the lipid A and LOS from *N. gonorrhoeae* (John et al., 1991; Yamasaki et al., 1991, 1994; Kerwood et al., 1992; Gibson et al., 1993) and the genes encoding the gonococcal GTase enzymes (Gotschlich, 1994; Erwin et al., 1996; Banerjee et al., 1998; John et al., 1999; Stein et al., 2011; Chakraborti et al., 2016) have led to a consensus around the prototypical LOS structures as illustrated in Figure 1. We aimed to use MALDI-TOF MS to profile and compare the structures of the LOS of the human challenge strains MS11 and FA1090.

Only a few groups have reported MS analysis of intact LOS (Dzieciatkowska et al., 2007; O'Brien et al., 2014). We have utilized a method for analysis of intact LOS by negative-ion MALDI-TOF MS using a thin-layer matrix composed of THAP and nitrocellulose to create an envelope of singly charged (M-H)^−^ molecular ions and prompt fragments to profile LOS mixtures without chemical modification (Sturiale et al., 2005; John et al., 2009a,b, 2012; Kilar et al., 2013). In this method, LOS molecular ions readily undergo “prompt” fragmentation, a type of in-source decay occurring at the sample surface in a few picoseconds to nanoseconds before or during desorption (Hoffman et al., 2007), to give fragments arising from the oligosaccharide and lipid A domains of the molecule through cleavage at the labile ketosidic linkage (Gibson et al., 1997; Sturiale et al., 2005). The prompt fragments representing both domains of the molecule corroborate the proposed compositions of the LOS molecular ions detected.

**Figure 2:**
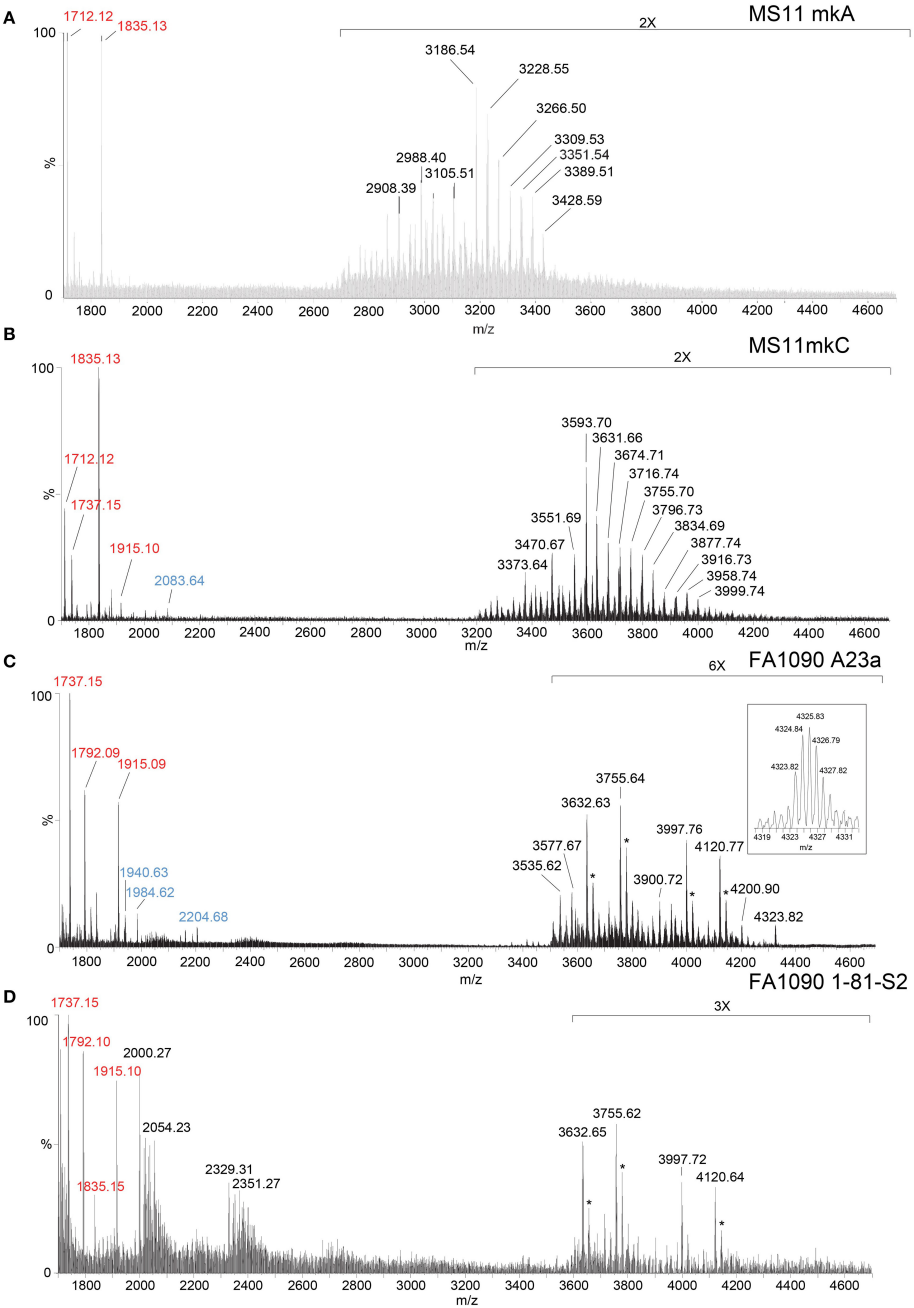
High-resolution negative-ion MALDI-TOF spectra of LOS from MS11mkA **(A)**, MS11mkC **(B)**, FA1090 A23a **(C)**, and FA1090 1-81-S2 **(D)** strains of *N. gonorrhoeae* purified using the PE method. Peaks for deprotonated molecular ions (M-H)^−^ are labeled in black font, lipid A prompt fragments in red font, and oligosaccharide prompt fragments in blue font. Peaks marked with an asterisk are for sodiated species.

We have shown that analysis of intact LOS in a high-resolution reflectron mode of MALDI-TOF MS to measure monoisotopic molecular ions with good mass accuracy (<20 ppm) increases the utility of the information that can be obtained compared to analysis in the linear low-resolution mode (Sturiale et al., 2011; John et al., 2012, 2016; Stephenson et al., 2013). Negative-ion MALDI-TOF spectra (Figure 2, Tables 1, 2) were obtained of intact LOS from MS11mkA, MS11mkC, FA1090 A23a, and FA1090 1-81-S2 (Figures 2A–D) with high resolution as illustrated in the inset (Figure 2C). There were several peaks observed with mass accuracies >20 ppm, which is likely due to an irregular peak shape or the presence of multiple components with the same nominal mass. The spectrum of the MS11mkA LOS was consistent with a structure consisting of a lactose (Galβ1-4Glc) α-chain (Figures 1, 2A), which was previously reported based on NMR and immunostaining of LOS after *O*-deacylation and dephosphorylation (Kerwood et al., 1992) and MS analysis of *O*-deacylated LOS (John et al., 1999). The MS analysis of the intact LOS presented here enabled the detection of peaks consistent with the substitution of some of the OS molecules with a α-chain consisting of GlcNAc with an *O*-acetyl group (Table 1). The single PEA on the oligosaccharide could be either 3- or 6-*O*-linked to HepII based on previously reported analyses of gonococcal and meningococcal LOS (O'Connor et al., 2006, 2008).

**Table 1:** Monoisotopic masses and proposed compositions for molecular ions and lipid A and oligosaccharide fragment ions from LOS of *N. gonorrhoeae* MS11 strains purified using the PE method.

**Strain**	***m/z***	**Derived Composition^a^**	**Calc *m/z***	**Δ PPM**
MS11mkA	3,428.487	TPLA PEA; 2 Kdo, 2 Hep, 3 Hex, HexNAc	3,428.574	−25.4
	3,389.511	TPLA PEA; 2 Kdo, 2 Hep, 2 Hex, HexNAc, PEA	3,389.529	−5.3
	3,351.544	DPLA PEA; 2 Kdo, 2 Hep, 2 Hex, HexNAc, PEA, OAc	3,351.573	−8.7
	3,309.529	DPLA PEA; 2 Kdo, 2 Hep, 2 Hex, HexNAc, PEA	3,309.563	−10.3
	3,266.501	TPLA PEA; 2 Kdo, 2 Hep, 2 Hex, HexNAc	3,266.521	−6.1
	3,228.550	DPLA PEA; 2 Kdo, 2 Hep, 2 Hex, HexNAc, OAc	3,228.565	−4.6
	3,186.542	DPLA PEA; 2 Kdo, 2 Hep, 2 Hex, HexNAc	3,186.554	−3.8
	3,105.505	DPLA; 2 Kdo, 2 Hep, 2Hex, HexNAc, OAc	3,105.557	−16.7
	2,988.401	DPLA PEA; 2 Kdo, 2 Hep, 2 Hex, HexNAc–(C_12_H_22_O_2_)	2,988.392	3.0
	1,835.125	DPLA PEA	1,835.126	−0.5
	1,737.148	TPLA PEA–(H_4_P_2_O_7_)	1,737.149	−0.6
	1,712.118	DPLA	1,712.118	0.0
	1,515.435	2 Kdo, 2 Hep, 2 Hex, 2 HexNAc, PEA, OAc	1,515.440	−3.3
	1,473.432	2 Kdo, 2 Hep, 2 Hex, 2 HexNAc, PEA	1,473.429	2.0
	1,295.374	2 Kdo, 2 Hep, 2 Hex, 2 HexNAc, OAc	1,295.381	−5.4
	1,253.367	Kdo, 2 Hep, 2 Hex, 2 HexNAc, PEA	1,253.371	−3.2
MS11mkC	3,999.742	TPLA PEA; 2 Kdo, 2 Hep, 3 Hex, 3 HexNAc, PEA, OAc	3,999.751	−2.3
	3,996.730	TPLA PEA; 2 Kdo, 2 Hep, 4 Hex, 3 HexNAc	3,996.785	−13.8
	3,958.737	DPLA PEA; 2 Kdo, 2 Hep, 4 Hex, 3 HexNAc, OAc	3,958.829	−23.2
	3,916.733	DPLA PEA; 2 Kdo, 2 Hep, 4 Hex, 3 HexNAc	3,916.819	−22.0
	3,877.743	DPLA PEA; 2 Kdo, 2 Hep, 3 Hex, 3 HexNAc, PEA	3,877.775	−8.3
	3,834.685	TPLA PEA; 2 Kdo, 2 Hep, 3 Hex, 3 HexNAc	3,834.732	−12.3
	3,796.731	DPLA PEA; 2 Kdo, 2 Hep, 3 Hex, 3 HexNAc, OAc	3,796.777	−12.1
	3,793.667	TPLA PEA; 2 Kdo, 2 Hep, 4 Hex, 2 HexNAc	3,793.706	−10.3
	3,755.695	DPLA PEA; 2 Kdo, 2 Hep, 4 Hex, 2 HexNAc, OAc	3,755.750	−14.6
	3,716.736	DPLA PEA; 2 Kdo, 2 Hep, 3 Hex, 2 HexNAc, PEA, OAc	3,716.706	8.1
	3,674.708	DPLA PEA; 2 Kdo, 2 Hep, 3 Hex, 2 HexNAc, PEA	3,674.695	3.5
	3,631.655	TPLA PEA; 2 Kdo, 2 Hep, 3 Hex, 2 HexNAc	3,631.653	0.6
	3,593.699	DPLA PEA; 2 Kdo, 2 Hep, 3 Hex, 2 HexNAc, OAc	3,593.697	0.6
	3,551.692	DPLA PEA; 2 Kdo, 2 Hep, 3 Hex, 2 HexNAc	3,551.687	−1.4
	3,470.665	DPLA 2 Kdo, 2 Hep, 3 Hex, 2 HexNAc, OAc	3,470.689	6.9
	2,083.642	2 Kdo, 2 Hep, 3 Hex, 3 HexNAc, PEA, OAc	2,083.651	4.3
	1,915.099	TPLA PEA	1,915.101	1.0
	1,880.575	2 Kdo, 2 Hep, 3 Hex, 2 HexNAc, PEA, OAc	1,880.572	−1.6
	1,835.132	DPLA PEA	1,835.127	−2.7
	1,737.149	TPLA PEA–(H_4_P_2_O_7_)	1,737.149	0.0
	1,712.116	DPLA	1,712.118	1.2
	1,660.519	Kdo, 2 Hep, 3 Hex, 2 HexNAc, PEA, OAc	1,660.515	−2.4

^a^Abbreviations for LOS structural moieties: TPLA, triphosphorylated lipid A; DPLA, diphosphorylated lipid A; Kdo, 3-deoxy-D-*manno*-octulosonic acid; Hep, heptose; Hex, hexose; HexNAc, *N*-acetylhexosamine; P, phosphate; PEA, phosphoethanolamine; OAc, *O*-linked acetate; Neu5Ac, *N*-acetylneuraminic acid.

**Table 2:** Monoisotopic masses and proposed compositions for molecular ions and lipid A and oligosaccharide fragment ions from LOS of *N. gonorrhoeae* FA1090 purified using the PE method.

**Strain**	***m/z***	**Derived composition^a^**	**Calc *m/z***	**Δ PPM**
A23a	4,323.815	TPLA PEA; 2 Kdo, 2 Hep, 5 Hex, 3 HexNAc, PEA, OAc	4,323.857	−9.7
FA1090	4,200.893	TPLA PEA; 2 Kdo, 2 Hep, 5 Hex, 3 HexNAc, OAc	4,200.848	10.7
	4,120.768	TPLA PEA; 2 Kdo, 2 Hep, 5 Hex, 2 HexNAc, PEA, OAc	4,120.778	−2.4
	4,078.768	TPLA PEA; 2 Kdo, 2 Hep, 5 Hex, 2 HexNAc, PEA	4,078.767	0.2
	3,997.763	TPLA PEA; 2 Kdo, 2 Hep, 5 Hex, 2 HexNAc, OAc	3,997.769	−1.5
	3,900.718	TPLA PEA–(H_4_P_2_O_7_); 2 Kdo, 2 Hep, 5 Hex 2 HexNAc, PEA	3,900.824	−27.2
	3,755.640	TPLA PEA; 2 Kdo, 2 Hep, 4 Hex, HexNAc, PEA, OAc	3,755.653	−3.5
	3,632.631	TPLA PEA; 2 Kdo, 2 Hep, 4 Hex, HexNAc, OAc	3,632.637	−1.7
	3,577.673	TPLA PEA–(H_4_P_2_O_7_); 2 Kdo, 2 Hep, 4 Hex HexNAc, PEA, OAc	3,577.702	−8.1
	3,535.623	TPLA PEA–(H_4_P_2_O_7_); 2 Kdo, 2 Hep, 4 Hex HexNAc, PEA	3,535.692	−19.5
	2,204.679	2 Kdo, 2 Hep, 5 Hex 2 HexNAc, PEA, OAc	2,204.677	0.9
	1,984.616	Kdo, 2 Hep, 5 Hex 2 HexNAc, PEA, OAc	1,984.619	−1.5
	1,940.628	Kdo, 2 Hep, 5 Hex 2 HexNAc, PEA, OAc–CO_2_	1,940.629	−0.5
	1,915.093	TPLA PEA	1,915.092	−0.5
	1,835.127	DPLA PEA	1,835.126	−0.5
	1,792.085	TPLA	1,792.084	−0.6
	1,737.149	TPLA PEA–(H_4_P_2_O_7_)	1,737.149	0.0
1-81-S2	4,120.643	TPLA PEA; 2 Kdo, 2 Hep, 5 Hex, 2 HexNAc, PEA, OAc	4,120.778	−32.8
FA1090	3,997.716	TPLA PEA; 2 Kdo, 2 Hep, 5 Hex, 2 HexNAc, OAc	3,997.769	−13.3
	3,755.620	TPLA PEA; 2 Kdo, 2 Hep, 4 Hex, HexNAc, PEA, OAc	3,755.653	−8.8
	3,632.647	TPLA PEA; 2 Kdo, 2 Hep, 4 Hex, HexNAc, OAc	3,632.637	2.8
	1,915.104	TPLA PEA	1,915.092	6.3
	1,835.149	DPLA PEA	1,835.126	12.5
	1,792.096	TPLA	1,792.084	6.7
	1,737.154	TPLA PEA–(H_4_P_2_O_7_)	1,737.149	2.9

^a^Abbreviations for LOS structural moieties: TPLA, triphosphorylated lipid A; DPLA, diphosphorylated lipid A; Kdo, 3-deoxy-D-*manno*-octulosonic acid; Hep, heptose; Hex, hexose; HexNAc, *N*-acetylhexosamine; P, phosphate; PEA, phosphoethanolamine; OAc, *O*-linked acetate; Neu5Ac, *N*-acetylneuraminic acid.

In the MALDI-TOF spectrum of intact MS11mkA LOS, prominent peaks could be observed for fragment ions of the phosphoforms of lipid A. The most prominent was the peak for 2 P PEA lipid A fragment ions at m/z 1,835.13, which was similar in size to that for diphosphoryl lipid A (2 P lipid A) ions at m/z 1,712.12. There was also a minor peak at m/z 1,737.15 that is for triphosphoryl lipid A with a single PEA (3 P PEA lipid), which has undergone facile fragmentation to lose H_4_P_2_O_7_ as we previously reported (John et al., 2009b). The most prominent lipid A fragment ion peaks were in accordance with the observation of peaks for highly abundant ions at m/z 3,186.54 and m/z 3,228.55 for intact LOS with 2 P PEA lipid A substituents and at m/z 3,105.51 for intact LOS with 2 P lipid A. The peaks for intact LOS at m/z 3,389.51 and m/z 3,266.50 are consistent with the expression of 3 P PEA lipid A.

### High-resolution MALDI-TOF MS profiling of MS11mkC LOS

Peaks for (M-H)^−^ ions (Table 1, Figure 2B) of MS11mkC were detected consistent with LOS molecules having 2 Kdo and 2 Hep along with one of the following sets of substituents: (i) 4 Hex, 3 HexNAc at m/z 3,996.73; (ii) 3 Hex, 3 HexNAc, PEA, and OAc at m/z 3,999.74, and 3 Hex, 3 HexNAc at m/z 3,834.69; and (iii) 3 Hex, 2 HexNAc, PEA, and OAc with 2 P PEA lipid at m/z 3,716.74, and 3 Hex, 2 HexNAc with 3 P PEA lipid A at m/z 3,631.66. These data are in accordance with the results of a prior analysis, which showed that MS11mkC expressed LOS with a HexNAcγ chain and an α-chain only that was mainly composed of 1 of 3 different groups as follows: (i) Galβ1-4GlcNAcβ1-4Galβ1-4GlcNAcβ1-3Galβ1-4Glcβ1, (ii) GalNAcβ1-3Galβ1-4GlcNAcβ1-3Galβ1-4Glcβ1, and (iii) Galβ1-4GlcNAcβ1-3Galβ1-4Glcβ1 and, thus, oligosaccharide compositions of 2 Kdo, 2 Hep, and 0-1 PEA groups with 4 Hex, 3 HexNAc; 3 Hex, 3 HexNAc; or 3 Hex, 2 HexNAc (John et al., 1999).

The most prominent of the (M-H)^−^ peaks for the MS11mkC LOS (Figure 2B) was observed at m/z 3,593.70 for 2 P PEA lipid A with 2 Kdo, 2 Hep, 3 Hex, 2 HexNAc, and OAc. Observed but of low abundance were peaks for fragment ions for the oligosaccharide moiety. For example, peaks consistent with 2 Kdo, 2 Hep, 3 Hex, PEA, and OAc with 3 HexNAc were observed at m/z 2,083.64 or with 2 Kdo, 2 Hep, 3 Hex, PEA, and OAc, and only 2 HexNAc at m/z 1,880.58. Interestingly, the relative abundance of the more highly phosphorylated or phosphoethanolaminylated lipid A molecules with 3 P PEA (m/z 1,915.10 and 1,737.15) and 2 P PEA (m/z 1,835.13) compared to 2 P lipid (m/z 1,712.12) was greater in the MS11mkC spectrum compared to the spectrum of MS11mkA.

### High-resolution MALDI-TOF MS profiling of FA1090 A23a and 1-81-S2 LOS

The same four major peaks were detected for (M-H)^−^ ions of FA1090 A23a and 1-81-S2 LOS in the MALDI-TOF MS spectra as presented in Figures 2C, D and listed in Table 2. These peaks were observed in the spectra at (i) m/z 4,120.77 and m/z 4,120.64, (ii) m/z 3,997.76 and m/z 3,997.72, (iii) m/z 3,755.64 and m/z 3,755.62, and (iv) m/z 3,632.63 and m/z 3,632.65, respectively. The peaks at m/z 4,120 differed from the peaks at m/z 3,997 by PEA (123 Da). Similarly, the peaks at m/z 3,755 differed by 123 Da from the peaks at m/z 3,632. The higher mass pairs of peaks at m/z 4,120 and m/z 3,997 differed from the lower mass pairs at m/z 3,755 and 3,622 by a Hex HexNAc (365 Da). The peaks at m/z 3,632 were consistent with a 3 P PEA lipid A with 2 Kdo, 2 Hep, 4 Hex, HexNAc, and OAc. The relative abundance of these four major peaks was very similar in the spectra of the A23a and the 1-81-S2 LOS. Both of the spectra also have peaks for sodiated (M-H_2_+Na)^−^ ions of the set of four peaks listed above.

There was a small peak observed in the spectrum of the A23a LOS at m/z 4,323.82 that is consistent with an additional HexNAc from the ions observed at m/z 4,120.77, which is in accordance with a composition of 3 P PEA lipid A with 2 Kdo, 2 Hep, 5 Hex, 3 HexNAc, PEA, and OAc. The m/z 4,323.82 peak was not observed in the spectrum of the LOS from the 1-81-S2 isolate, but given the low abundance of the ions, this is likely due to differences in sample purity, loading, or matrix spot-to-spot variability.

Of prominence in the FA1090 1-81-S2 spectrum is a set of non-LOS peaks at m/z 2,000.27 and m/z 2,329.31 with related peaks that differ by the addition of 16 Da and 22 Da. These also occur but are much less readily apparent in the spectrum of the FA1090 A23a LOS.

The compositional information that we derived from the high-resolution MS spectra presented in Figure 2 coupled with the previously reported immunoblot data (Erwin et al., 1996; Hobbs et al., 2013) indicates that the four most common LOS of the FA1090 LOS has the conserved 2 Kdo, 2 Hep core with the *O*-acetylated GlcNAc γ-chain, a Galβ1-4Glc (lactosyl) β-chain, and an α-chain composed of either Galβ1-4Glc (lactosyl) or Galβ1-4GlcNAcβ1-3Galβ1-4Glc (LNnT) with or without a PEA group on the oligosaccharide. Thus, the compositions are consistent with those deduced from the high-resolution MS data (Figure 2) as presented in Table 2. The higher mass peak in the spectrum of the A23a LOS at m/z 4,323.82 was in accordance with the expression of the GalNAc added by the gonococcal LgtD GTase onto the non-reducing terminus of the LNnT moiety and fitting with the aforementioned SDS-PAGE and mAb 2C7 immunoblot analyses.

Although the relative abundance of peaks for oligosaccharide fragment ions in the spectra is low, those observed support the compositions derived from the (M-H)^−^ peaks for the intact LOS. For example, in the spectrum of the A23a variant (Figure 2C, Table 2), peaks are observed at m/z 2,204.68 and m/z 1,984.62 that are consistent with a Kdo, 2 Hep, 5 Hex, 2 HexNAc, PEA, and OAc moiety with and without the second Kdo (220 Da).

The lipid A fragment ions detected show that the major form present is substituted with 3 P and PEA, as indicated by the prominent peaks at m/z 1,915.1 and m/z 1,737.2. Peaks were also observed for 2 P PEA lipid A at m/z 1,835.2 and 3 P lipid A at m/z 1,792.1. However, unlike the spectra of MS11mkA and mkC, there was no significant peak for 2 P lipid A ions at m/z 1,712.1.

### MALDI-TOF MS of LOS from an *eptA* mutant of 1-81-S2 FA1090

**Figure 3:**
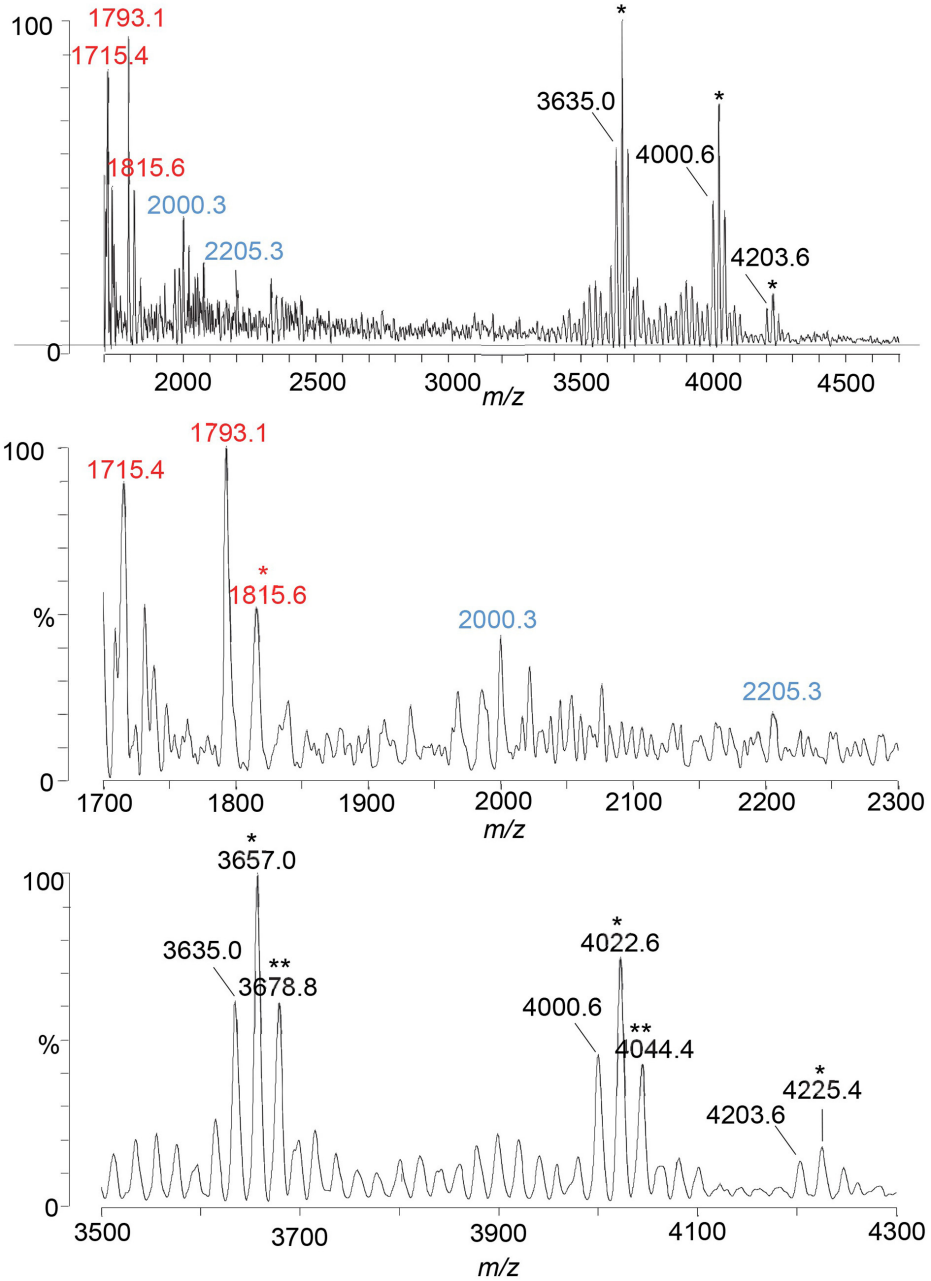
A spectrum from negative-ion MALDI-TOF MS in the linear mode is shown of the LOS from the *eptA* mutant of the FA1090 1-81-S2 strain of *N. gonorrhoeae* that was purified using the PE method. Peaks for deprotonated molecular ions (M-H)^−^ are labeled in black font, lipid A prompt fragments in red font, and oligosaccharide prompt fragments in blue font. Peaks marked with an asterisk are for sodiated species.

A spectrum was acquired of intact LOS from the *eptA* mutant of 1-81-S2 (Figure 3, Table 3) lacking PEA on the lipid A to aid in distinguishing substitution of the 1-81-S2 LOS with P-PEA groups (202.975 Da monoisotopic mass) from HexNAc substituents (203.079 Da monoisotopic mass). The LOS was analyzed on the Axima MALDI-TOF MS in linear mode and, therefore, is of low resolution.

**Table 3:** Average masses and proposed compositions for molecular ions and lipid A and oligosaccharide fragment ions of LOS from an *eptA* mutant of *N. gonorrhoeae* FA1090 1-81-S2 purified using the PE method.

**Strain**	***m/z***	**Derived composition^a^**	**Calc *m/z***	**Δ Da**
1-81-S2	4,203.6	TPLA; 2 Kdo, 2 Hep, 5 Hex, 3 HexNAc, OAc, PEA	4,203.3	0.3
Δ*EptA*	4,000.6	TPLA; 2 Kdo, 2 Hep, 5 Hex, 2 HexNAc, OAc, PEA	4,000.1	0.5
FA1090	3,635.0	TPLA; 2 Kdo, 2 Hep, 4 Hex, HexNAc, OAc, PEA	3,634.8	0.2
	2,205.3	2 Kdo, 2 Hep, 5 Hex, 2 HexNAc, PEA, OAc	2,205.9	−0.6
	2,000.3	2 Kdo, 2 Hep, 4 Hex 2 HexNAc PEA	2,001.7	−1.4
	1,815.6	TPLA, Na	1,815.2	0.4
	1,793.1	TPLA	1,793.2	−0.1
	1,715.4	DPLA	1,713.2	2.2

^a^Abbreviations for LOS structural moieties as in Table 1.

Peaks for intact LOS were detected at m/z 4,000.6 for TPLA; 2 Kdo, 2 Hep, 5 Hex, 2 HexNAc, OAc, and PEA, and for a loss of Hex HexNAc at m/z 3,635.0 for TPLA; 2 Kdo, 2 Hep, 4 Hex, HexNAc, OAc, and PEA, which were also observed in the spectrum of the LOS from the wild-type 1-81-S2. Furthermore, a peak was observed for an LOS with an additional HexNAc at m/z 4,203.6 for TPLA; 2 Kdo, 2 Hep, 5 Hex, 3 HexNAc, OAc, and PEA. The analogous peak with the additional PEA on the lipid A moiety was detected in the high-resolution spectrum of the A23a LOS at m/z 4,323.82 but not in the spectrum of the wild-type 1-81-S2 LOS (Figure 2), likely because the latter sample was less abundant and produced less signal with more abundant background peaks. The peaks for oligosaccharide fragment ions at m/z 2,205.3 and m/z 2,000.3 (Figure 3) provided additional support for the compositions proposed above for the FA1090 LOS in Table 2.

The most prominent peak for lipid A is that observed at m/z 1,793.1 for 3 P lipid A, which is in accordance with the peaks observed in the spectrum of the wild-type 1-81-S2 LOS (Figure 2D) that had fragment ions for 3 P PEA and 3 P PEA lipid A-H_4_P_2_O_7_. In the spectrum of the LOS of the *eptA* mutant, a less prominent peak was also observed at m/z 1,715.4 for 2 P lipid A.

### MALDI-TOF MS of MS11mkC LOS isolated by ME enzyme digestion and treated with HF

A goal in the *Neisseria* field is to profile intact LOS that is directly isolated from gonococci that have invaded human cells or infected people without propagating the bacteria *in vitro*, which would better enable the correlation of LOS structure with virulence and infection. Achieving this goal will require highly sensitive methods of both purification and detection of LOS. We have obtained good sensitivity in MALDI-TOF MS profiling of intact LOS with 2–5 μg (~0.5–1 pmol) loaded per spot, although spot-to-spot variability in preparation of the thin-layer matrix to date has often meant that loading multiple spots was required. Two groups have described breakthroughs in the analysis of intact LOS using LC coupled with ESI-MS that enabled obtaining multiple MS/MS spectra from 1-μg samples of LOS (O'Brien et al., 2014; Pupo et al., 2021). However, without further refinement, the classical method for the isolation of LOS from *Neisseria* and other Gram-negative bacteria, which uses sequential enzymatic digestion followed by phenol-water extraction, does not enable the purification of LOS from small numbers of bacteria. Therefore, we evaluated a method that utilizes ME digestion that was first described for the isolation of LOS from relatively small numbers of *C. jejuni* and, furthermore, requires less time than the PE method (Dzieciatkowska et al., 2008).

**Figure 4:**
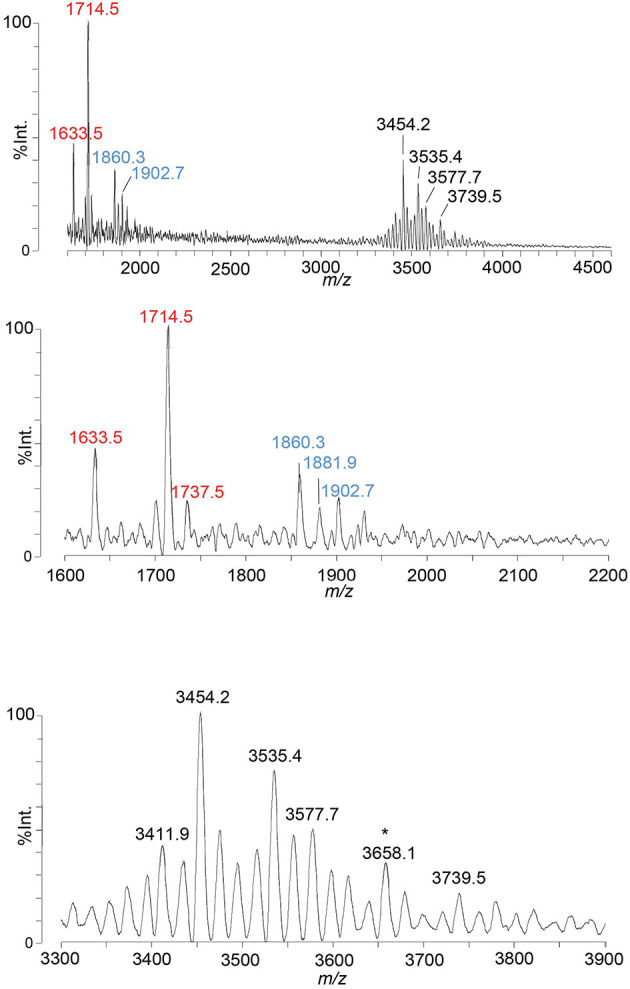
A spectrum from negative-ion MALDI-TOF MS in the linear mode is shown of MS11mkC LOS purified using the ME method and treated with HF. Peaks for deprotonated molecular ions (M-H)^−^ are labeled in black font, lipid A prompt fragments in red font, and oligosaccharide prompt fragments in blue font. The peak marked with an asterisk is for sodiated species.

We applied the ME method to the isolation of the LOS from the well-characterized MS11mkC, which was subsequently treated with HF to cleave phosphodiester bonds that occur on the lipid A to reduce the heterogeneity of the LOS and simplify the resultant spectrum (Figure 4). Proposed compositions for the major peaks in the spectrum are presented in Table 4. Peaks were detected at m/z 3,739.5 for the LOS with 2 P lipid A with only a single Kdo moiety and 2 Hep, 4 Hex, 3 HexNAc, PEA, and OAc, and at m/z 3,577.7 for the LOS that differs by a Hex, and m/z 3,535.4 that also lost the OAc group. The LOS peaks were consistent with the oligosaccharide fragment ion peaks at m/z 1,902.7, m/z 1,881.9, and m/z 1,860.3. Importantly, the spectral quality and peaks observed are similar to those we have observed previously in MALDI-TOF MS of HF-treated LOS purified using the classical method of enzyme digestion with PE (John et al., 2009b).

**Table 4:** Average masses and proposed compositions for molecular ions and lipid A and oligosaccharide fragment ions from *N. gonorrhoeae* LOS MS11mkC purified using the ME method and HF-treated.

**Strain**	***m/z***	**Derived composition^a^**	**Calc *m/z***	**Δ Da**
MS11mkC	3,739.5	DPLA; Kdo, 2 Hep, 4 Hex, 3 HexNAc, PEA, OAc	3,741.0	−1.5
	3,577.7	DPLA; Kdo, 2 Hep, 3 Hex, 3 HexNAc, PEA, OAc	3,579.0	−1.3
	3,535.4	DPLA; Kdo, 2 Hep, 3 Hex, 3 HexNAc, PEA	3,536.8	−1.2
	3,454.2	MPLA; Kdo, 2 Hep, 3 Hex, 3 HexNAc, PEA	3,456.8	−2.6
	3,411.9	MPLA; Kdo, 2 Hep, 3 Hex, 3 HexNAc, PEA–CO_2_	3,412.8	−0.9
	1,902.7	Kdo, 2 Hep, 4 Hex, 3 HexNAc, OAc	1,903.7	−1.0
	1,881.9	2 Kdo, 2 Hep, 3 Hex, 2 HexNAc, PEA, OAc	1,881.6	−0.3
	1,860.3	Kdo, 2 Hep, 4 Hex, 3 HexNAc, OAc, PEA	1,859.7	0.6
	1,737.5	TPLA PEA–(H_4_P_2_O_7_)	1,738.3	−0.8
	1,714.5	DPLA	1,713.2	1.3
	1,633.5	MPLA	1,633.2	0.3

^a^Abbreviations for LOS structural moieties as in Table 1.

### Comparison of MALDI-TOF MS profiling of FA1090 A23a LOS isolated by PE and ME enzyme digestion

**Figure 5:**
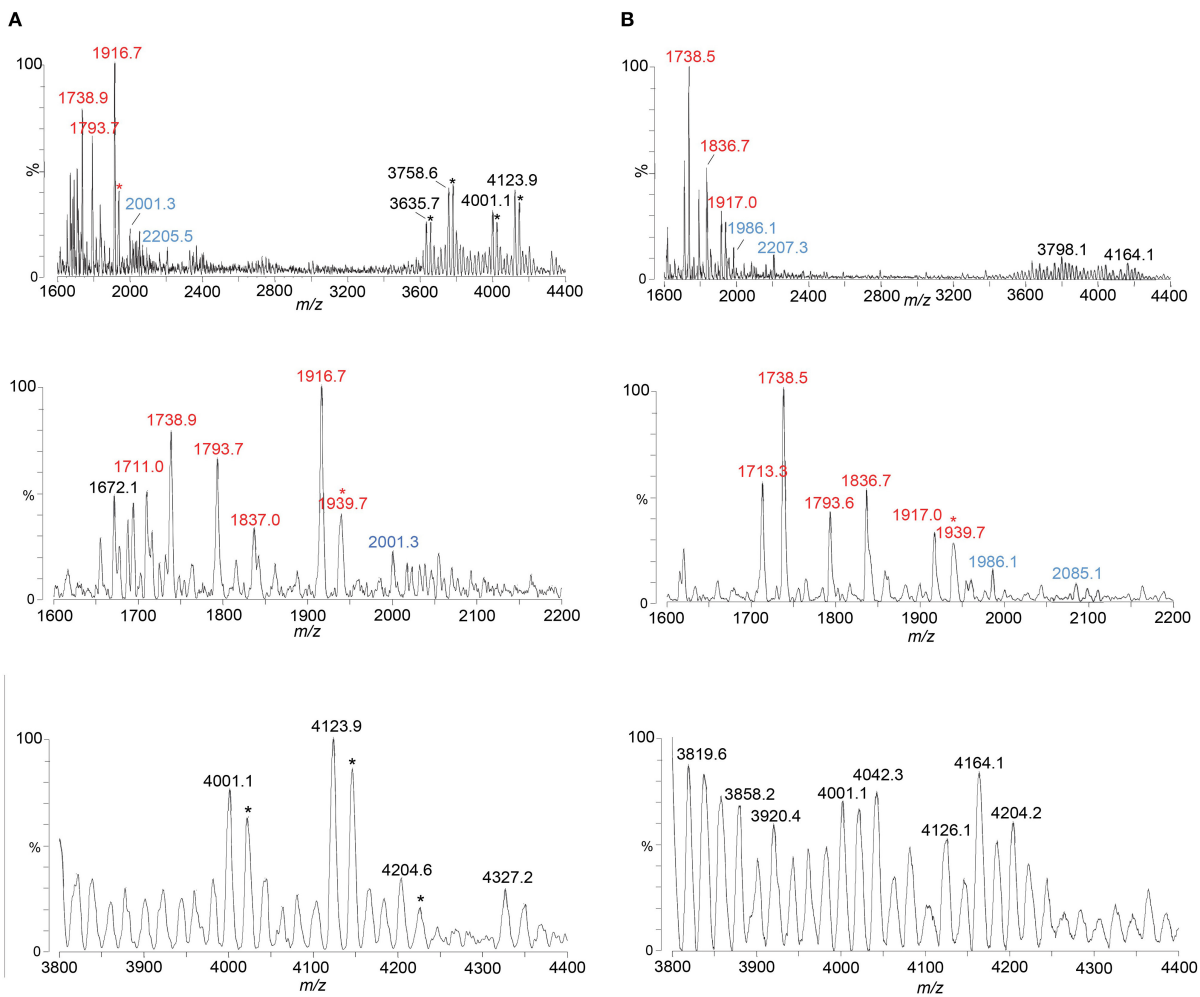
Spectra from negative-ion MALDI-TOF MS in the linear mode are shown of FA1090 A23a gonococci purified using the PE method **(A)** and using the ME method **(B)**. Peaks for deprotonated molecular ions (M-H)^−^ are labeled in black font, lipid A prompt fragments in red font, and oligosaccharide prompt fragments in blue font. Peaks marked with an asterisk are for sodiated species.

We used the ME method to isolate LOS from FA1090 A23a and compared its spectrum to that of the LOS isolated using the classical PE method, both acquired in the linear negative-ion mode on the Axima MALDI-TOF MS. The compositions proposed for the peaks observed (Figures 5A, B) are presented in Table 5. The spectra had all four of the most prominent peaks observed when the LOS was analyzed on the Synapt HDMS (Figure 2C). However, the prominence of the peaks differed, particularly in the spectrum of the LOS purified using the ME method (Figure 5B). Peaks were observed (Figures 5A, B) at m/z 4,123.9 and m/z 4,126.1, which were consistent with 3 P PEA lipid A with the 2 Kdo 2 Hep core, α-chain HexNAc OAc, and 5 Hex 2 HexNAc PEA. The peaks at m/z 4,123.9 and m/z 4,126.1 differed by a PEA (123 Da) from the peaks at m/z 4,001.1. The peaks at m/z 4,123.9 and m/z 4,126.1 differed by Hex HexNAc (365 Da) from the peaks at m/z 3,758.6, and by Hex HexNAc PEA (488 Da) from the peaks at m/z 3,635.7 and m/z 3,636.6, respectively. The compositions proposed (Table 5) for the ions are in accordance with the presence of the same heterogeneity in the LOS molecules in both preparations.

**Table 5:** Average masses and proposed compositions for molecular ions, and lipid A and oligosaccharide fragment ions from *N. gonorrhoeae* FA1090 A23a LOS purified using the PE (top) and ME (bottom) methods.

**Strain**	***m/z***	**Derived composition^a^**	**Calc *m/z***	**Δ Da**
A23a	4,327.2	TPLA PEA; 2 Kdo, 2 Hep, 5 Hex, 3 HexNAc, PEA, OAc	4,326.3	0.8
FA1090	4,123.9	TPLA PEA; 2 Kdo, 2 Hep, 5 Hex, 2 HexNAc, PEA, OAc	4,123.1	0.8
	4,001.1	TPLA PEA; 2 Kdo, 2 Hep, 5 Hex, 2 HexNAc, OAc	4,000.1	1.0
	3,758.6	TPLA PEA; 2 Kdo, 2 Hep, 4 Hex, HexNAc, PEA OAc	3,757.8	0.8
	3,635.7	TPLA PEA; 2 Kdo, 2 Hep, 4 Hex, HexNAc, OAc	3,634.7	0.9
	2,205.5	2 Kdo, 2 Hep, 5 Hex, 2 HexNAc, PEA, OAc	2,205.9	1.5
	2,001.3	2 Kdo, 2 Hep, 4 Hex 2 HexNAc, PEA	2,001.7	−0.4
	1,939.7	TPLA PEA Na	1,938.2	1.4
	1,916.7	TPLA PEA	1,916.3	0.4
	1,793.7	TPLA	1,793.2	0.4
A23a	1,738.9	TPLA PEA–(H_4_P_2_O_7_)	1,738.3	0.6
	4,204.2	TPLA PEA; 2 Kdo, 2 Hep, 5 Hex, 3 HexNAc, OAc	4,203.3	0.9
FA1090	4,164.1	TPLA PEA; 2 Kdo, 2 Hep, 5 Hex, 3 HexNAc	4,161.2	2.8
	4,126.1	TPLA PEA; 2 Kdo, 2 Hep, 5 Hex, 2 HexNAc, PEA, OAc	4,123.1	3.0
	4,042.3	TPLA PEA; 2 Kdo, 2 Hep, 4 Hex, 3 HexNAc, OAc	4,041.1	1.2
	4,001.1	TPLA PEA; 2 Kdo, 2 Hep, 5 Hex, 2 HexNAc, OAc	4,000.1	1.0
	3,798.1	DPLA; 2 Kdo, 2 Hep, 5 Hex, 2 HexNAc, OAc	3,797.1	1.0
	3,758.6	TPLA PEA; 2 Kdo, 2 Hep, 4 Hex, HexNAc, PEA OAc	3,757.8	0.8
	3,636.6	TPLA PEA; 2 Kdo, 2 Hep, 4 Hex, HexNAc, OAc	3,634.7	1.8
	2,207.3	2 Kdo, 2 Hep, 5 Hex, 2 HexNAc, PEA, OAc	2,205.9	1.5
	2,085.1	2 Kdo, 2 Hep, 5 Hex, 2 HexNAc, OAc	2,082.8	2.2
	1,986.1	Kdo, 2 Hep, 5 Hex 2 HexNAc, PEA, OAc	1,985.7	0.4
	1,917.0	TPLA PEA	1,916.3	0.7
	1,836.7	DPLA PEA	1,793.2	0.5
	1,793.6	TPLA	1,793.2	0.4
	1,738.5	TPLA PEA–(H_4_P_2_O_7_)	1,738.3	0.2
	1,713.3	DPLA	1,713.2	0.1

^a^Abbreviations for LOS structural moieties as in Table 1.

Several peaks for oligosaccharide fragment ions were observed (Figures 5A, B). These were seen at m/z 2,205.5 and m/z 2,207.3, fitting with a composition of 2 Kdo, 2 Hep, 5 Hex, 2 HexNAc, PEA, and OAc. The loss of PEA from the peak at m/z 2,207.3 (Figure 5B) was consistent with the ions at m/z 2,085.1.

Most of the same suite of peaks for lipid A fragment ions were observed in the high-resolution spectrum of the A23a LOS acquired on the Synapt (Figure 2C) as when the LOS was purified using the PE and ME methods and analyzed on the Axima (Figures 5A, B). The major peaks observed were for 3 P PEA lipid A at m/z 1,917, 3 P lipid A at m/z 1,794, and 3 P PEA lipid A that lost H_4_P_2_O_7_ (178 Da) at m/z 1,739. The relative abundance of the 3 P PEA lipid A fragment ions was greater in the low-resolution spectra, whereas the relative abundance of the 3 P PEA—H_4_P_2_O_7_ lipid A fragment ions was greater in the high-resolution spectrum (Figure 2C).

### MALDI-TOF MS profiling of FA1090 1-81-S2 LOS isolated by ME enzyme digestion of bacteria cultured with and without CMP-Neu5Ac

Sialylation of LOS is known to be an important mechanism of defense for gonococci against complement-mediated lysis (Ram et al., 1998; McQuillen et al., 1999; Wu and Jerse, 2006), but the dynamics of sialylation have not been followed during the course of infection because studies have depended on indirect methods such as the lack of binding of anti-LOS oligosaccharide epitope antibodies (McGee et al., 1996; Schneider et al., 1996). Toward the development of new methods for the analysis of LOS sialylation, we profiled LOS from FA1090 1-81-S2 that was purified by ME enzyme digestion and analyzed by MALDI-TOF MS after culture with and without supplementation with the sialic acid donor substrate CMP-Neu5Ac. *N. gonorrhoeae* does not produce the donor substrate CMP-Neu5Ac for sialylation; therefore, sialylation of Lst-expressing *N. gonorrhoeae* requires a source of CMP-conjugated Neu5Ac, whether in culture medium or in the infection milieu.

**Figure 6:**
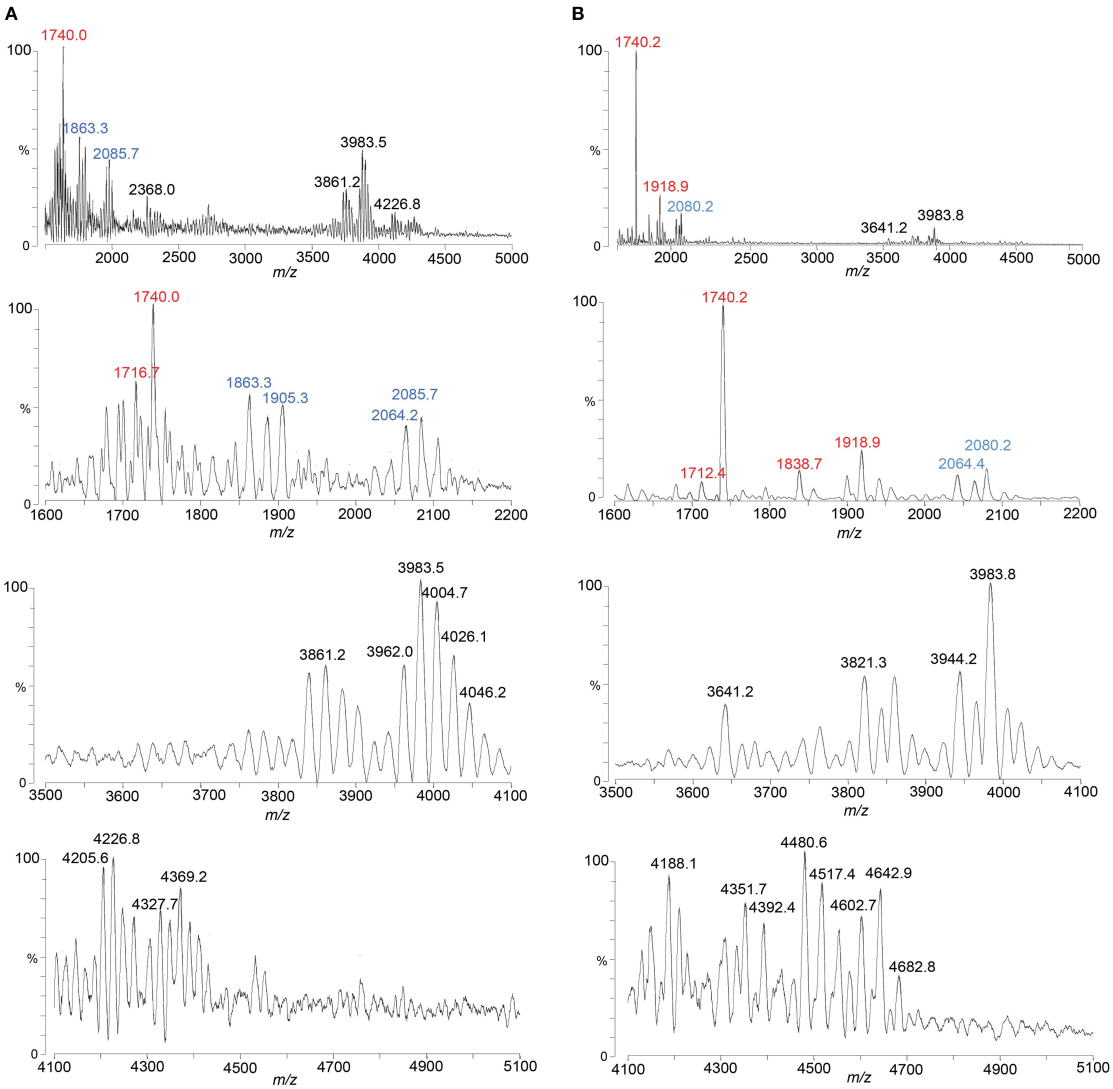
Spectra from negative ion MALDI-TOF MS in the linear mode are shown of LOS from 1-81-S2 FA1090 gonococci grown with **(B)** and without **(A)** CMP-Neu5Ac-containing media and purified using the ME method. Peaks for deprotonated molecular ions (M-H)^−^ are labeled in black font, lipid A prompt fragments in red font, and oligosaccharide prompt fragments in blue font.

**Figure 7:**
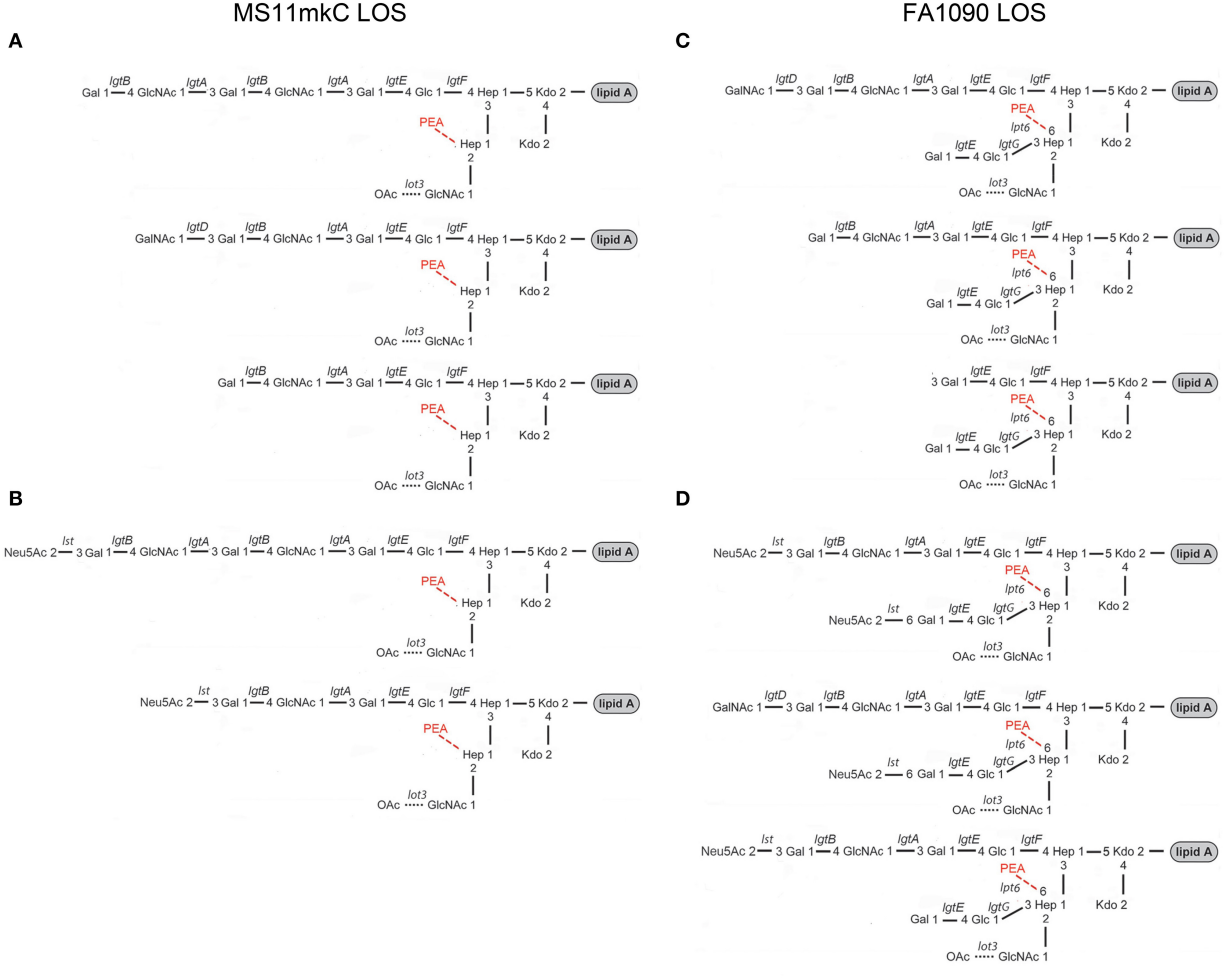
Major structures of the MS11mkC **(A)** and FA1090 1-81-S2 **(C)** LOS are consistent with published data (20–24) and the mass spectra presented herein. Postulated structures of the **(B)** sialylated LOS from MS11mkC bacteria after growth in the presence of Neu5Ac are in accordance with the specificity of the Neisserial Lst enzyme (80–82). Major structures of the monosialylated and disialylation LOS from FA1090 1-81-S2 **(D)** are consistent with the mass spectrum obtained after the growth of bacteria on GC media containing CMP-Neu5Ac (Figure 6, Table 6) and supported by data on the Lst-catalyzed sialylation of non-reducing terminal lactose on HepII (37, 38).

The spectrum of the 1-81-S2 LOS (Figure 6A, Table 6) isolated using the ME method from bacteria cultured on media without CMP-Neu5Ac had a peak at m/z 4,369.2 (Figure 6A) that is consistent with a 6 Hex and 3 HexNAc oligosaccharide. This composition is in accordance with a (LacNAc)_2_-Lac α-chain on HepI, as illustrated in Figure 7A, and we reported it for MS11mkC (John et al., 1999) with a Lac α-chain on HepII. Peaks at m/z 4,327.7 and m/z 4,205.6 differed by a PEA and were consistent with the 2 Kdo, 2 Hep, 5 Hex, 3 HexNAc oligosaccharide moiety that was the largest detected in the high-mass resolution spectra of the LOS from either the A23a or the 1-81-S2 variants (Figures 2C, D, Table 2).

**Table 6:** Average masses and proposed compositions for *N. gonorrhoeae* LOS FA1090 1-81-S2 grown with (bottom) and without (top) CMP-Neu5Ac and purified using the ME method.

**Strain**	***m/z***	**Derived composition^a^**	**Calc *m/z***	**Δ Da**
1-81-S2	4,369.2	TPLA PEA; 2 Kdo, 2 Hep, 6 Hex, 3 HexNAc, OAc	4,365.4	3.8
FA1090	4,327.7	TPLA PEA; 2 Kdo, 2 Hep, 5 Hex, 3 HexNAc, PEA, OAc	4,326.3	1.4
	4,226.8	TPLA PEA; 2 Kdo, 2 Hep, 5 Hex, 3 HexNAc, OAc, Na	4,225.3	1.5
	4,205.6	TPLA PEA; 2 Kdo, 2 Hep, 5 Hex, 3 HexNAc, OAc	4,203.3	2.3
	4,004.7	TPLA PEA; 2 Kdo, 2 Hep, 3 Hex, 3 HexNAc, PEA, OAc	4,002.0	2.7
	3,983.5	TPLA PEA; Kdo, 2 Hep, 5 Hex, 3 HexNAc, OAc	3,983.1	0.4
	3,962.0	TPLA PEA; 2 Kdo, 2 Hep, 3 Hex, 3 HexNAc, PEA	3,960.0	2.0
	3,861.2	TPLA PEA; 2 Kdo, 2 Hep, 4 Hex, 2 HexNAc, OAc, Na	3,859.9	1.3
	3,839.7	TPLA PEA; 2 Kdo, 2 Hep, 4 Hex, 2 HexNAc, OAc	3,837.9	1.8
	2,085.7	Kdo, 2 Hep, 5 Hex, 3 HexNAc, OAc, Na	2,087.8	−2.0
	2,064.2	Kdo, 2 Hep, 5 Hex, 3 HexNAc, OAc	2,065.8	−1.6
	1,863.3	Kdo, 2 Hep, 5 Hex, 2 HexNAc, OAc	1,862.6	0.7
	1,740.0	TPLA PEA–(H_4_P_2_O_7_)	1,738.3	1.7
1-81-S2	4,682.8	TPLA PEA; 2 Kdo, 2 Hep, 5 Hex, 2 HexNAc, PEA, 2 Neu5Ac, Na	4,685.6	−2.8
FA1090	4,642.9	TPLA PEA; 2 Kdo, 2 Hep, 5 Hex, 3 HexNAc, OAc, PEA, Neu5Ac, Na	4,639.6	3.3
	4,602.7	DPLA PEA; 2 Kdo, 2 Hep, 5 Hex, 2 HexNAc, PEA, 2 Neu5Ac, Na	4,605.6	−2.9
	4,517.4	TPLA PEA; 2 Kdo, 2 Hep, 5 Hex, 3 HexNAc, OAc, Neu5Ac, Na	4,516.5	0.9
	4,480.6	DPLA PEA; 2 Kdo, 2 Hep, 5 Hex, 2 HexNAc, 2 Neu5Ac, Na	4,482.6	−2.0
	4,392.4	TPLA PEA; 2 Kdo, 2 Hep, 5 Hex, 2 HexNAc, PEA, Neu5Ac, Na	4,394.5	−2.1
	4,351.7	TPLA; 2 Kdo, 2 Hep, 5 Hex, 2 HexNAc, OAc, Neu5Ac, Na	4,348.3	3.4
	4,188.1	TPLA PEA; 2 Kdo, 2 Hep, 4 Hex, 3 HexNAc, OAc, PEA, Na	4,190.3	1.9
	3,983.8	TPLA PEA; Kdo, 2 Hep, 5 Hex, 3 HexNAc, OAc	3,983.1	1.8
	3,944.2	TPLA PEA; Kdo, 2 Hep, 4 Hex, 3 HexNAc, OAc, PEA	3,944.0	0.2
	3,821.3	TPLA PEA; Kdo, 2 Hep, 4 Hex, 3 HexNAc, OAc	3,821.0	0.3
	3,641.2	TPLA PEA; Kdo, 2 Hep, 4 Hex, 3 HexNAc, OAc–(H_4_P_2_O_7_)	3,643.0	−1.8
	2,080.2	2 Kdo, 2 Hep, 5 Hex, 2 HexNAc, OAc	2,082.8	−2.6
	2,064.4	Kdo, 2 Hep, 5 Hex, 3 HexNAc, OAc	2,065.8	−1.4
	1,918.9	Kdo, 2 Hep, 4 Hex, 3 HexNAc, OAc	1,916.3	2.6
	1,838.7	DPLA PEA	1,836.3	2.4
	1,740.2	TPLA PEA–(H_4_P_2_O_7_)	1,738.3	1.9

^a^Abbreviations for LOS structural moieties as in Table 1.

There were several peaks in common between the spectra of the LOS isolated from bacteria cultured on media without and with CMP-Neu5Ac (Figures 6A, B). Notably, the most prominent LOS peaks in the spectra are at m/z 3,983.5 and m/z 3,983.8, consistent with a TPLA PEA LOS having an oligosaccharide with 5 Hex, 3 HexNAc, and OAc, and a core oligosaccharide that has lost one of the two Kdo moieties. This composition is also fitting with the observation of peaks in the spectra at m/z 2,064.2 and m/z 2,064.4 for oligosaccharide fragment ions with a composition of Kdo, 2 Hep, 5 Hex, 3 HexNAc, and OAc.

The spectrum of the 1-81-S2 LOS (Figure 6B, Table 6) isolated using the ME method from bacteria cultured on media with CMP-Neu5Ac differed significantly in the higher mass region from the spectrum of LOS isolated from bacteria grown without CMP-Neu5Ac. There were a number of peaks observed in the spectrum of the LOS isolated from bacteria grown on CMP-Neu5Ac-containing media (Figure 6B) at greater m/z than the peaks at m/z 4,369.2 and m/z 4,327.7, which were peaks of the highest m/z values in the spectrum of the LOS grown on media without CMP-Neu5Ac supplementation (Figure 6A). These higher mass peaks were observed at m/z 4,682.8, m/z 4,642.9, m/z 4,602.7, m/z 4,517.4, m/z 4,480.6, and m/z 4,392.4 and were consistent with sodium adducts of LOS containing one or even two Neu5Ac moieties (Table 6). The extent of the sodium adduction may not be surprising given the highly negatively charged nature of the LOS, which has 2-3 phosphoryl groups, 2 Kdo, and 1 or 2 Neu5Ac monosaccharides.

As illustrated in Figure 1B, the LgtD-catalyzed GalNAc substitution of terminal Gal of the LNnT group on HepI is in competition with α-2,3 sialylation of the LOS. However, no other GTase is thought to compete with the α-2-6 sialylation of the terminal Gal of the lactosyl group on HepII. Accordingly, the compositions fitting the observed peaks in this spectrum have up to 3 HexNAcs with a single Neu5Ac that would be expected to be on the lactosyl group on HepII. There were only 2 HexNAcs in compositions consistent with two Neu5Acs, which could be on the terminal Gal of the LNnT moiety on HepI and the terminal Gal of the lactosyl group on HepII, as shown in Figure 7D. Although in other strains substitution at either of these positions has been reported (Gulati et al., 2005; Ram et al., 2018), sialylation of both the α-chain LNnT on HepI and the β-chain lactosyl group on HepII of LOS, as appears to occur on the FA1090 LOS, has not been described previously.

## Discussion

The goals of our study were to characterize the FA0190 A23a and 1-81-S2 LOS in comparison with the MS11mkA and mkC strains and to determine the applicability of the ME method to the purification of Neisserial LOS from a small number of bacteria. Unlike the clear differences we confirmed and have previously reported in the oligosaccharide structures of the LOS expressed by the MS11mkA and MS11mkC human challenge strains (John et al., 1999), there were no obvious changes in the LOS expressed by the 1-81-S2 FA1090 strain compared to the A23a inoculum FA1090 strain. There have been numerous previous studies of the structures of gonococcal LOS using mass spectrometry, immunomolecular profiling, and NMR. Furthermore, the genes encoding for and the specific glycosyltransferases and other enzymes responsible for the biosynthesis of the LOS have been identified and characterized. These data have enabled the definition of a suite of prototypical gonococcal LOS structures (Figure 1) that are anticipated to be presented within a context for potential differences in the structures due to phase-variable gene expression. Given the prototypical structures that have been reported for the LOS of *N. gonorrhoeae* (Figure 1), the published data showing the immunochemical recognition of the FA1090 LOS by 3F11 and 2C7 mAbs, the monosaccharide composition of the FA1090 LOS determined from high pH anion exchange profiles (Erwin et al., 1996), and the MS profiles of the A23a and 1-81-S2 LOS that we present herein, the major structural features for these isolates (Figure 7) are as follows: a highly-phosphorylated lipid A with 3 P PEA lipid A being the predominant structure, a conserved 2 Kdo, 2 Hep core structure, the presence of α-, β-, and γ-chains on the oligosaccharide, with the HepII α-chain comprising a lactosyl moiety, the HepII β-chain GlcNAc being at least partially *O*-acetylated, and a HepI γ-chain LNnT partially substituted with a non-reducing terminal GalNAc. The four major peaks in the spectra were consistent with the substitution of the oligosaccharide with 0–1 PEA groups, which, when present, would be expected to be *O*-6 linked because there is the β-chain lactosyl group that is *O*-3 linked to HepII (Yamasaki et al., 1999; Gulati et al., 2005).

The prominence of peaks in the A23a and 1-81-S2 spectra (Figure 2) at m/z 3,632.6 and m/z 3,755.6 are consistent with a composition of TPLA PEA; 2 Kdo, 2 Hep, 4 Hex, and OAc, and only 1 HexNAc with 0 or 1 PEA groups, suggests that these represent a smaller LOS rather than only being fragment ions of the larger LOS observed at m/z 3,997.7 and m/z 4,120.7 with a composition of TPLA PEA; 2 Kdo, 2 Hep, 5 Hex, and OAc with 2 HexNAcs and 0 or 1 PEA groups. The presence of smaller LOS with both α- and β-chains is supported by the previously reported immunoblot showing 2C7 recognition of FA1090 LOS that is similar in size to that of the 15323 strain, which has lactose moieties on both HepI and HepII (Yamasaki et al., 1999; Gulati et al., 2005).

A difference that we observed between the two MS11 strains was that MS11mkA expressed relatively more abundant ions for 2P lipid A. We also observed variation between the spectra of the FA1090 and the MS11 strains in the relative abundance of ions for peaks for phosphoforms of lipid A. The MS11 strains expressed relatively more ions for 2 P lipid A, 2 P PEA lipid A, and fewer ions for 3 P PEA lipid A. Previous analyses have shown that more lipid A substitution with P and PEA increases the induction of inflammatory cytokines such as TNF-α and, furthermore, that greater PEA substitution is correlated with increased binding to the complement regulatory protein C4BP and diminished killing by normal serum (John et al., 2009b; Lewis et al., 2009, 2013; Liu et al., 2014). Thus, the relatively lower lipid A phosphorylation is in accordance with the MS11 strains being moderately serum-sensitive and the FA1090 strains being highly serum-resistant (Carbonetti et al., 1990; Chen and Seifert, 2013).

The α2-3 sialylation of terminal Gal on the α-chain LNnT LOS reduces the sensitivity of *N. gonorrhoeae* to complement-mediated killing in serum, at least partly by increasing its binding to human factor H (Ngampasutadol et al., 2008; Lewis et al., 2015). However, α2-6 sialylation of the terminal Gal on the HepII β-chain lactose or the alternative trisaccharide α-chain (Galα1-3Galβ1-4Glcβ1-4HepI) did not increase binding of the LOS to factor H but did confer some resistance to complement-mediated killing (Gulati et al., 2005; Ram et al., 2018). Furthermore, in the female mouse model of genital tract infection, mutant gonococci functionally deficient in sialyltransferase activity had reduced infectivity (Wu and Jerse, 2006). Conversely, it was shown in the controlled human urethral infection model that increased sialylation of the LOS of *N. gonorrhoeae* significantly reduced infectivity in men (Schneider et al., 1996). Evidence suggests that female-to-male transmission of infection is aided by the desialylation of sialylated LOS by sialidases in cervicovaginal secretions that are apparently of bacterial origin (Ketterer et al., 2016).

There were 1-3 HexNAc monosaccharides in the compositions fitting the FA1090 spectra. These data are consistent with the presence of a single GlcNAc on the γ-chain, the GlcNAc within LNnT (Galβ1-4GlcNAcβ1-3Galβ1-4Glcβ1), and the partial substitution of LNnT with a non-reducing GalNAc catalyzed by the gonococcal LgtD GTase. Our analyses of the spectrum of the 1-81-S2 LOS from bacteria grown on media supplemented with CMP-Neu5Ac are in accordance with up to 2 Neu5Ac groups in the presence of 2 but not 3 HexNAc moieties, which is consistent with α-2,3 sialylation of the terminal Gal on the α-chain LOS and α-2-6 sialylation of the terminal Gal of the lactosyl group on the β-chain. Furthermore, if the FA1090 LOS has the potential to be disialylated, it would be anticipated to have a higher level of sialylation overall than LOS from gonococcal isolates such as MS11mkC, which does not express the β-chain lactosyl group. Thus, we postulate that the much greater infectivity of MS11 compared to FA1090 could be at least partly due to the greater sialylation of the latter strain (Hobbs et al., 2011).

Comparing the spectra of LOS purified using the PE compared to the ME method, there was a good signal-to-background ratio in the spectrum of the HF-treated MS11mkC LOS purified by ME. Furthermore, the (M-H)^−^ peaks detected for the LOS and lipid A and oligosaccharide prompt fragment ions were consistent with our analysis (Figure 2) of the intact LOS purified by PE (John et al., 1999).

There were differences in the relative abundances of peaks in the spectra of the intact LOS from FA1090 A23a purified by the PE and ME methods (Figures 5A, B, Table 5). Nonetheless, there were numerous peaks in common, such as those for the apparent major species TPLA PEA; 2 Kdo, 2 Hep, 4 Hex, HexNAc, and OAc (calculated m/z 3,634.7), TPLA PEA; 2 Kdo, 2 Hep, 4 Hex, HexNAc, PEA, and OAc (calculated m/z 3,757.8), and TPLA PEA; 2 Kdo, 2 Hep, 5 Hex, 2 HexNAc, PEA, and OAc (calculated m/z 4,123.1). Thus, despite the differences in the relative prominence of some peaks in these two spectra, overall, our results strongly support the potential utility of the ME enzymatic purification protocol for the preparation of LOS for MS analysis.

The reduction in time and the reagents required are additional advantages of the ME compared to the PE method for isolation of the LOS. The extensive 1- to 2-day dialysis performed with the PE method to remove traces of phenol also reduces the concentration of sodium chloride and other salts in the media or buffer solutions. Although we added a microdialysis step to the ME purifications we performed, the presence of more salt adducts, such as (M+Na-2H)^−^, in the spectra of intact LOS, which is most apparent in the spectrum of the sialylated intact LOS (Figure 6), likely was the major reason for the lower signal-to-noise ratios and differences in relative prominence of some peaks. Increasing the length of time for the dialysis should help reduce the levels of salt. The signal-to-noise ratios in the spectra that we acquired in the past of HF-treated LOS purified using the PE method were very similar to those in the spectrum (Figure 4) of the HF-treated LOS purified using the ME method. This is likely due to the reduction in the number of peaks in the sample due to dephosphorylation, which also reduced the anionic nature of the sample and, therefore, its salt adducts.

## Conclusion

Our analyses suggest that there are several differences in the LOS structures of MS11mkC and FA1090 strains that could be determinants of the dramatic difference in infectivity of the two strains, specifically the increased lipid A phosphorylation, expression of a 6-linked PEA, and the potential for sialylation at two different sites on the FA1090 LOS. We expect that the application of the ME method for purifying the LOS on a microscale method, when coupled with other recently described methods for LC-MS/MS (Pupo et al., 2021), will enable the characterization of the phenotype and sialylation of the phase-variable gonococcal LOS from bacteria isolated from within human cells or hosts without expansion of the bacterial population *in vitro*. Toward this goal, we are currently exploring the use of affinity chromatography with mAb to the LOS to isolate *N. gonorrhoeae* from human cells.

## Data availability statement

The original contributions presented in the study are included in the article/supplementary material, further inquiries can be directed to the corresponding author.

## Author contributions

CJ, AKC, and GJ: conceptualization. CJ: methodology and formal analysis. CJ and NP: investigation. AJC and AKC: resources. CJ: writing—original draft. CJ, NP, AJC, AKC, and GJ: writing—review and editing. NP: visualization. GJ: supervision. AKC and GJ: funding acquisition. All authors contributed to the article and approved the submitted version.
